# Nutrient transfer and antioxidant effect of adzuki bean before and after GABA enrichment

**DOI:** 10.3389/fnut.2023.1123075

**Published:** 2023-01-26

**Authors:** Xiujie Jiang, Qingpeng Xu, Jiayu Zhang, Zhijiang Li, Huacheng Tang, Dongmei Cao, Dongjie Zhang

**Affiliations:** ^1^College of Food Science, Heilongjiang Bayi Agricultural University, Daqing, China; ^2^National Coarse Cereals Engineering Research Center, Heilongjiang Bayi Agricultural University, Daqing, China

**Keywords:** adzuki beans, germination, GABA, nutritional changes, antioxidant activity

## Abstract

In order to study the nutritional changes of γ-aminobutyric acid (GABA) enrichment in adzuki bean germination, vacuum combined with monosodium glutamate (MSG) was used as the germination stress of adzuki bean. The nutrient transfer before and after GABA enrichment in adzuki bean germination under vacuum combined with MSG stress were studied by means of chromatography and scanning electron microscope (SEM). The antioxidant activity and hypoglycemic effect of different solvent extracts before and after germination of adzuki bean were evaluated by experiments in vitro. The results showed that the nutritional characteristics of adzuki bean rich in GABA changed significantly (*P* < 0.05), the total fatty acids decreased significantly (*P* < 0.05), and the 21 amino acids detected increased significantly. After germination, the starch granules of adzuki bean became smaller and the surface was rough Germination stress significantly increased the antioxidant and hypoglycemic activities of the extracts from different solvents (*P* < 0.05), and the water extracts had the best effect on DPPH and ⋅OH radical scavenging rates of 88.52 and 83.56%, respectively. The results indicated that the germinated adzuki bean rich in GABA was more nutritious than the raw adzuki bean and had good antioxidant activity. It hoped to provide technical reference for rich food containing GABA.

## 1. Introduction

Adzuki bean is an important dietary bean, which originated in China and has been cultivated for about 12,000 years. It is mainly distributed in East Asian countries including Japan, South Korea, and China (1, 2). Adzuki bean is rich in protein, dietary fiber and minerals, and has a moderate proportion of amino acids, among which lysine up to 1.8% is recognized as a healthy food source (3). In addition, adzuki bean also contains bioactive components with health enhancing characteristics, including polyphenols anthocyanin saponins, which endowed adzuki bean with good edible and medicinal value (4, 5). Many studies *in vivo* and *in vitro* have confirmed that adzuki bean and its water extract have physiological effects such as antioxidant properties (3), reducing fat accumulation and insulin resistance induced by high-fat diet (6, 7), inhibiting muscle atrophy (8).

γ-aminobutyric acid is a free-form tetracarbon non-protein amino acid, abbreviated as GABA, which widely exists in microorganisms, plants and vertebrates. It is the main inhibitory neurotransmitter in the brain and plays an important role in brain metabolism (9). In recent years, with the deepening of research, many physiological functions such as anti-anxiety, calming nerves and lowering blood pressure of γ-aminobutyric acid (GABA) have been revealed continuously, and GABA has good thermal stability and safety, which has become a potential new food functional factor and plays an irreplaceable role in the regulation of life activities (10). However, the content of GABA in higher plants is very low, only 0.03–2.00 μ mol/g, so the amount of GABA ingested by human body from natural food cannot meet the physiological needs of normal body. At the same time, with the increase of age and mental stress in life, the content of GABA in human body will decrease day by day (11). Therefore, how to increase the content of GABA in seeds and foods of cereals and beans has become a research hotspot. It is more important for human health to obtain natural foods with high GABA content.

It is worth noting that germination can significantly increase the GABA content in beans (12), and adzuki beans contain high levels of glutamate (13), which has the potential to enrich GABA through germination stress, which is helpful to improve the nutritional and functional characteristics of adzuki beans and experience the health benefits brought by GABA-rich adzuki beans diet. At the same time, the theoretical research on the enrichment of GABA in adzuki bean is weak, and there are no reports on nutrition transfer and hypoglycemic *in vitro* during the enrichment of GABA in adzuki bean. In this study, the changes and transfer of main nutrients and functional nutrients before and after germination and enrichment of GABA in adzuki bean were analyzed, and different solvents were selected to extract effective components from germination adzuki bean to comprehensively evaluate the antioxidant activity of different solvent extracts from adzuki bean rich in GABA. It provides theoretical basis for developing edible high GABA adzuki bean food in real life.

## 2. Materials and methods

### 2.1. Materials and chemicals

Adzuki bean (Vigna angularis) raw materials (variety, Pearl Red) were purchased from Daqing Ruizefeng Technology Co., Ltd., (Daqing, China) and stored in a low temperature environment. GABA was purchased from Sigma Co., Ltd. (Shanghai, China). Methanol with purity of 99.9% was purchased from Thermo Scientific (China) Co., Ltd. (Shanghai, China). Anhydrous sodium sulfate and N-hexane with purity of 99.9% were purchased from Sinopharm Chemical Reagent Co., Ltd. (Shanghai, China). The standard fatty acid methyl ester is purchased from Sigma Co., Ltd. (Shanghai, China), in which the mother liquor was stored at −20°C. The working solution is now used and prepared. Twenty-two kinds of amino acid standards was purchased from Beijing Solarbio Technology Co., Ltd. (Beijing, China). Other drugs in the experiment are analytical pure grade, and purchased from local reagent companies.

### 2.2. Method of adzuki beans enriched GABA by germination technology

#### 2.2.1. Soaking treatment

Adzuki bean was rinsed, then soaked in 0.1% sodium hypochlorite solution for 15 min, then rinsed with ionized water for 3 times, added with 4 times volume of purified water, adjusted to pH 5 with citric acid and disodium hydrogen phosphate solution, soaked at 35°C for 16 h.

#### 2.2.2. Germination treatment

After soaking, the adzuki beans were washed with deionized water and spread evenly on the germination board of four layers of gauze. Put it in a vacuum drying oven, opened the vacuum pump, vacuumize to −0.1 MP, and set the temperature at 35°C, closed the vacuum pump, germinated under vacuum stress for 16 h, and then transferred it to a constant temperature oxygen incubator for normal germination until 48 h. Sprayed 2.0 mmol/L sodium glutamate solution every 1 h. In the whole germination process, the temperature should be kept at 35°C. The above-mentioned soaking and germination technology is based on the previous research results. According to this technology, the GABA content in adzuki bean was as high as 254.19 mg/100 g. Among them, vacuum combined with MSG optimizes the enrichment of GABA in adzuki bean and the determination of GABA content in adzuki bean. See the author’s literature (14).

### 2.3. Determination of basic nutrients

The nutritional components of germinated adzuki bean before and after GABA enrichment were detected. The basic nutrients such as protein (GB 5009.5-2016), fat (GB 5009.6-2016), starch (GB 31637-2016), and dietary fiber (GB/T 5009.88-2016) were all determined by the latest national standard method in China. Determination of mineral element content performed the operation according to the literature method (15).

### 2.4. Amino acid detection

Quantitative analysis of 22 amino acids in germinated adzuki bean and raw adzuki bean were carried out by LC-MS. The specific detection steps are as follows:

Configuration of standard koji: First, the single standard of 22 kinds of amino acids is weighed and prepared into corresponding mother liquor with methanol, and the mother liquor of the single standard is taken in equal amount to form mixed standard of amino acids, and then diluted to the required concentration with 10% formic acid, methanol and water in equal proportion to make the working standard liquid required for detection. Finally, the mother liquor and working liquid are stored at 4°C for subsequent application.

Sample treatment: Weighing the germinated adzuki bean and raw adzuki bean samples were placed in 2 mL EP tube, respectively, added 600 μL 10% formic acid and water in equal proportion, added several steel balls to shake for 30 s. After grinding for 90 s, centrifuged at low temperature and high speed for 5 min (12,000 rpm). Absorbed 100 μL supernatant and added it to the same volume internal standard. After 30 s of oscillation treatment, the filter solution is a high concentration detection solution by filtering and sterilizing with 0.22 μm membrane. Then the high concentration detection solution is diluted, which took 10 μL high concentration detection solution, added mixed solution with 99 times of 10% formic acid and water in equal proportion.

Using vortex oscillation for 30 s, absorbed 100 μL diluted solution and added equal volume isotope internal standard solution. Then oscillate, filter and sterilize again. At this time, the filtrate is a low concentration detection solution.

Chromatographic conditions: The liquid chromatographic column is Agilent C18 column (4.6 × 150 mm, the model of ZORBAX Eclipse XDB-C18), the injection amount is set to 5 μL, and the column temperature is selected at 40°C. The mobile phase A is 10% methanol water (containing 0.1% formic acid) and the mobile phase B is 50% methanol water (also containing 0.1% formic acid). The elution conditions are firstly washed with low concentration mobile phase B. Then flushed with 10–100% B between 0 and 7 min, used 100% B flushing between 7 and 18 min. After that, the concentration of mobile phase decreased slowly, within 18–18.5 min, flushed using 10–100% of mobile phase B, within 18.5–21 min, flushed using 10% of mobile phase B. The flowing rate is set for 0.3–0.4 mL/min.

Mass spectrometry conditions: Selecting the positive ion mode, ion source temperature was set at 500°C,voltage selected 5 500 V, collision gas for 6 psi, curtain gas for 30 psi, atomization gas and auxiliary gas were 50 psi, respectively (16).

### 2.5. Fatty acid detection

#### 2.5.1. Symbolic configuration

First, the mixed standard of 51 fatty acid methyl esters were diluted by gradient. The initial concentration of the mixed standard was 4 000 μg/mL, which was diluted with N-hexane to 1, 5, 10, 25, 50, 100, 250, 500, 1000, and 2000 μg/mL, respectively. The mixed standard solution needs to be frozen at −20°C. The working solution can be prepared according to need. The internal standard was methyl salicylate of 500 ppm.

#### 2.5.2. Sample preparation

Weighing the sprouted adzuki bean and raw adzuki bean samples in a 2 mL centrifuge tube, added the mixed solution (chloroform and methanol 2:1 ratio solution), added a certain amount of glass beads, oscillated with a special tissue grinder for 60 s, repeated the operation twice, ultrasonic extracted at 25°C for 30 min. Then centrifuged at low temperature and high speed for 5 min (12,000 rpm), absorbed the supernatant and transferred it to a 15 mL centrifuge tube. It added 2 mL 1% sulfuric acid and methanol solution by whirlpool vibration for uniform mixing. The esterification reaction was treated in water bath at 80°C for 30 min. After cooling, 1 mL N-hexane was added for extraction and mixing, and then kept for 5 min, then washed with cold water and centrifuged at low temperature and low speed for 10 min. The upper supernatant of 700 μL was absorbed and transferred to centrifuge tube, which removed the residual water in the sample with proper amount of anhydrous sodium sulfate, and then mix evenly, oscillate and centrifuge. It absorbed 100 μL of the above solution in a centrifuge tube and diluted it with 4 times the volume of N-hexane. After that, absorbed the treatment sample with 300 μL N-hexane, and then added 15 μL internal standard, and the mixed evenly for standby.

Absorb the upper 700 μL supernatant transfer centrifuge tube, remove the residual water in the sample with proper amount of anhydrous sodium sulfate, and then mix evenly, oscillate and centrifuge. Absorb 100 μL of the above solution in a centrifuge tube and dilute it with 4 times the volume of n-hexane; After that, the sample treated with 300 μL N-hexane was absorbed, and then added with 15 μL internal standard, and then mixed evenly for later using.

#### 2.5.3. Chromatographic conditions

Chromatographic column selected Thermo Fisher capillary, whose specification wad 50 m × 0. 25 mm ID × 0. 20 μm. The temperature from injection to transmission was 250, 300, and 280°C, respectively, when the injection volume was 1 μL/min. The detection carrier gas was helium gas, which flow rate was 0.63 mL/min.

#### 2.5.4. Mass spectrometry conditions

Pay attention to the selection of electron bombardment ionization (EI) source, scanning mode set to 70 eV electron energy in SIM environment. For more detailed information, referred to the method of Beccaria et al. (17).

### 2.6. Microstructure analysis

Scanning electron microscope was used to observe the microstructure of adzuki bean before and after germination, and proper amount of germinated adzuki bean powder was fixed on the sample table in a certain order, sprayed with gold and sent to the sample room. After adjusting the working voltage to 5.0 kV, the microstructure of adzuki bean powder was observed at times of 500, 2000, and 3500 k, respectively. Then selected clear and representative pictures to take pictures.

### 2.7. Determination of antioxidant indexes *in vitro*

(1) Sample extraction. The germinated adzuki bean and raw material adzuki bean are pulverized and sifted with 80 meshes. Water, ethanol, methanol, ethyl acetate, and N-hexane were selected as the extraction solution 0.50 g samples were weighed and added with 20 times extraction solution. Ultrasonic extraction was carried out at 45°C for 1 h, centrifuged at 4,500 rpm for 5 min, and filtrate and filter residue were collected respectively. The extraction solution such as water, ethanol, methanol, and N-hexane were concentrated under reduced pressure, then freeze-dried. Finally, the extracts and precipitates were stored in a refrigerator at −80°C for later using.
(2) 2,20-diphenyl-1-picrylhydrazyl (DPPH) clearance rate. It referred to the documentation method (18), 2 mL sample solution and 0.1 mmol/L DPPH solution were weighed, the two solutions are mixed in equal proportion. The mixed solution reacted at room temperature and away from light for 1 h. Then the absorbance was measured at 517 nm. It calculated the clearance rate according to formula (1).

(1) $$\mathrm{DPPH}\mathrm{clearance}\mathrm{rate}\left(\%\right)=\left[1-\frac{\left(A_{1}-{\text{A}}_{2}\right)}{{\text{A}}_{0}}\right]\times 100$$

The formula A_0_ represented the absorbance value of DPPH + ethanol solution. A_1_ indicated the absorbance value of DPPH + sample solution.

A_2_ indicated the absorbance value of sample + ethanol solution.

(3) ⋅OH clearance rate. It referred to the documentation method (18), absorbing 1 mL of sample extraction solution, added 1 mL of 6 mmol/L of FeSO_4_ solution, which added 1 mL of 6 mmol/L of salicylic acid-ethanol and H_2_O_2_ solution in turn, then reacted in water bath at 37°C for 30 min, then quickly measured the absorbance of each sample extraction solution at 510 nm.

### 2.8. Statistical analysis

The experimental data were analyzed by SPSS 19.0 (IBM Corp., NY, USA) software, and the data expressed as mean ± SD. ANOVA with Duncan’s multiple range tests were performed to evaluate the data significance of basic nutrition indexes. A value of *P* < 0.05 or 0.01 was considered statistically significant. Line graphs were illustrated using Graph Pad Prism 7.0 (San Diego, CA, USA).

## 3. Results

### 3.1. Effect of germination on basic components of adzuki bean

According to Table 1, the contents of protein, fat, and starch in adzuki bean were obviously changed after germination under vacuum and MSG stress for 48 h, which decreased by 15.42, 13.86, and 17.88%, respectively comparing with raw adzuki bean. This may be due to the activation of protease, lipase, and amylase in adzuki bean during germination, which leaded to the consumption of the original storage nutrients in adzuki bean as energy and the decomposition of some of them into small molecules by endogenous enzymes for the synthesis of new substances (19). In addition, the metabolic process of bean germination will have a certain impact on the content of mineral elements. This study showed that the Na, Ca, Mg element in adzuki bean increased significantly after germination (Table 2). This may be closely related to the degradation of phytic acid during germination. It is reported that the decrease of phytic acid content is more conducive to the synthesis of minerals such as calcium, iron and magnesium (20).

**TABLE 1:** Changes of main components of adzuki beans before and after germination (g/100 g).

Sample	Moisture	Ash	Fat	Protein	Dietary fiber	Starch
Adzuki beans	9.27 ± 2.15^a^	4.26 ± 1.12^a^	1.37 ± 0.06^a^	25.15 ± 1.04^a^	15.24 ± 2.62^a^	45.45 ± 3.58^a^
Germinated adzuki bean	11.34 ± 1.03^b^	4.21 ± 1.23^a^	1.18 ± 0.08^a^	21.27 ± 1.27^b^	18.4 ± 2.04^b^	37.32 ± 5.64^b^

^a^, ^b^letter indicated that there was significant difference between the same nutritional components of adzuki bean and germinated adzuki bean (*P* < 0.05).

**TABLE 2:** Changes of mineral elements in adzuki beans before and after germination (mg/kg).

Sample	P	Na	K	Ca	Mg
Adzuki beans	3290.80 ± 12.57^a^	1551.74 ± 20.45^a^	11048.26 ± 18.79^a^	1649.69 ± 30.45^a^	1516.77 ± 32.67^a^
Germinated adzuki bean	3141.42 ± 18.24^b^	1992.52 ± 22.18^b^	10271.13 ± 21.48^b^	2005.08 ± 19.67^b^	1583.33 ± 28.43^a^

^a^, ^b^letter indicated that there is significant difference between red bean and germinated adzuki bean in the same element (P < 0.05).

### 3.2. Effect of germination on fatty acid composition of adzuki bean

**FIGURE 1:**
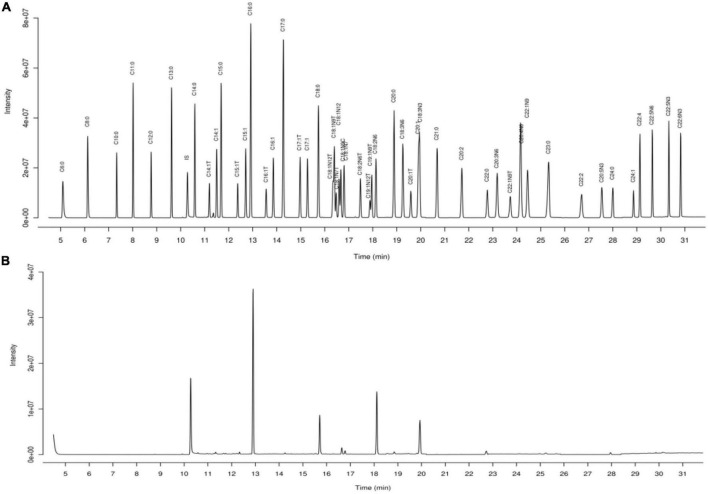
Total ion flow chromatogram of mixed standard **(A)** and sample **(B)**.

The fatty acid composition of sprouting adzuki bean and raw adzuki bean was quantitatively analyzed by gas chromatography-mass spectrometry (GC-MS), in which the total ion flow chromatogram of standard and sample is shown in Figure 1, and the quantitative analysis results of 51 fatty acids are shown in Table 3.

**TABLE 3:** Fatty acid composition of adzuki bean before and after germination.

Fatty acid	Abbreviations	Adzuki bean (μg/g)	Germinated adzuki bean (μg/g)
Caproate	C6:0	0.567	0.656
Caprylate	C8:0	0.386	0.425
Caprate	C10:0	0.419	0.499
Unndecanoate	C11:0	0.379	0.802
Laurate	C12:0	3.406	5.228
Tridecanoate	C13:0	0.353	0.474
Myristate	C14:0	11	16.158
Myristelaidate	C14:1T	10.691	5.72
Myristoleate	C14:1	5.853	5.146
Pentadecanoate	C15:0	8.146	11.31
10-Transpentadecenoate	C15:1T	34.465	36.649
10-Pentadecenoate	C15:1	10.044	8.163
Palmitate	C16:0	1690.353	1786.854
Palmitelaidate	C16:1T	5.521	1.753
Palmitoleate	C16:1	8.946	25.914
Heptadecanoate	C17:0	13.115	18.723
10-Transsheptadecenoate	C17:1T	9.025	9.212
10-Heptadecenoate	C17:1	11.465	12.746
Stearate	C18:0	448.605	505.751
Petroselaidate	C18:1N12T	1.309	ND
Elaidate	C18:1N9T	4.73	3.624
Transvaccenate	C18:1N7T	4.554	2.965
Petroselinate	C18:1N12	130.176	175.57
Oleate	C18:1N9C	136.942	107.125
Vaccenate	C18:1N7	85.037	97.445
Linoelaidate	C18:2N6T	1.888	1.696
7-Transnonadecenoate	C19:1N12T	3.477	2.103
10-Transnonadecenoate	C19:1N9T	7.252	6.064
Linoleate	C18:2N6	1411.753	1575.879
Arachidate	C20:0	25.683	30.462
Gamma linolenate	C18:3N6	3.255	4.288
*Trans*-11-eicosenoate	C20:1T	4.73	3.495
11-eicosenoate	C20:1	378.358	480.124
Alpha linolenate	C18:3N3	822.957	1032.511
Heneicosanoate	C21:0	5.631	7.956
11,14 eicosadienoate	C20:2	5.436	6.096
Behenate	C22:0	85.758	88.334
Homogamma linolenate	C20:3N6	2.12	2.079
Brassidate	C22:1N9T	9.842	8.055
Erucate	C22:1N9	16.284	15.38
11-14-17 Eicosatrienoate	C20:3N3	3.881	3.458
Arachidonate	C20:4N6	5.176	5.809
Tricosanoate	C23:0	13.235	17.211
Docosadienoate	C22:2	4.722	4.031
Eicosapentaenoate (EPA)	C20:5N3	1.399	1.776
Lignocerate	C24:0	46	84.246
Nervonoate	C24:1	10.779	8.173
Docosatetraenoate	C22:4	2.357	1.647
Docosapentaenoate (DPA)	C22:5N6	2.013	1.387
Docosapentaenoate	C22:5N3	1.566	0.937
Docosahexaenoate (DHA)	C22:6N3	1.203	2.261

It can be seen from Table 3 that the total fatty acid content in adzuki bean decreased from 5512.24 to 4658.49 μg/g before and after germination. The composition of saturated and unsaturated fatty acids between C6–C24 indicated that the total fatty acid content in adzuki bean changed but the types of fatty acids were not affected by germination stress. Further analysis showed that short chain fatty acids such as caproic acid, caprylic acid, decanoic acid, and lauric acid in germinated adzuki bean increased significantly and oleic acid content decreased significantly (*P* < 0.05). The content of essential fatty acids beneficial to human health increased significantly (*P* < 0.05). The content of linoleic acid and linolenic acid increased by 11.63 and 25.46%, respectively compared with that before germination and accounted for 55.99% of the total fatty acids. In addition, the content of arachidonic acid EPA, DHA, which had the function of anti-fatigue and promoting brain development, were also accumulated during germination.

### 3.3. Effect of germination on amino acid composition of adzuki bean

It can be seen from Table 4 that the total amino acid content of adzuki bean treated by vacuum combined with MSG increased from 2780.65 to 12343.93 μg/g, which increased by 4.44 times except homocysteine, the other 21 amino acids increased significantly (*P* < 0.05). The content of asparagine, glutamate, arginine was 4919.42, 2104.47, and 2150.97 μg/g, which was 10.78, 1.43, 3.14 times higher than that of the original adzuki bean, which had important contribution to the total amount of amino acids. It is worth noting that glutamine increased by 184.12 times after germination, which is mutually confirmed by the decrease of protein content in adzuki bean after germination. During the germination process, protein is decomposed into small molecular amino acids under the action of endogenous enzymes, thus increasing the nutritional components of germination products and being more beneficial to human digestion and absorption.

**TABLE 4:** Amino acid composition of adzuki beans before and after germination.

Amino acid	Abbreviations	adzuki bean (μg/g)	Germinated adzuki bean (μg/g)
Glycine	Gly	7.58	39.02
L-Alanine	Ala	88.39	1157.08
4-Aminobutyric acid	GABA	16.79	1140.69
L-Serine	Ser	165.02	1071.75
L-Proline	Pro	109.64	858.33
L-Valine	Val	77.31	1050.65
L-Threonine	Thr	5.36	132.47
L-Isoleucine	Ile	26.65	353.87
L-Leucine	Leu	23.23	346.69
L-Asparagine	Asn	455.95	4919.42
L-Ornithine hydrochloride	Orn	1.14	3.07
L-Aspartic acid	Asp	631.82	1034.02
DL-Homocysteine	Hcy	ND	ND
L-Glutamine	Gln	2.65	487.92
L-Lysine	Lys	150.22	1309.71
L-Glutamic acid	Glu	1469.24	2104.47
L-Methionine	Met	3.76	97.40
L-Histidine	His	101.12	881.12
L-Phenylalanine	Phe	109.08	733.98
L-Arginine	Arg	685.25	2150.97
L-Tyrosine	Tyr	52.58	420.58
L-Tryptophan	Trp	193.54	242.15

In a word, the nutritional characteristics of adzuki bean were significantly changed by vacuum combined with MSG stress from the changes of nutrients during the germination of adzuki bean. The general trend is to be more conducive to increasing the nutritional value of adzuki bean, regulating physiological activities and benefiting human health.

### 3.4. Effect of germination on microstructure of adzuki bean

**FIGURE 2:**
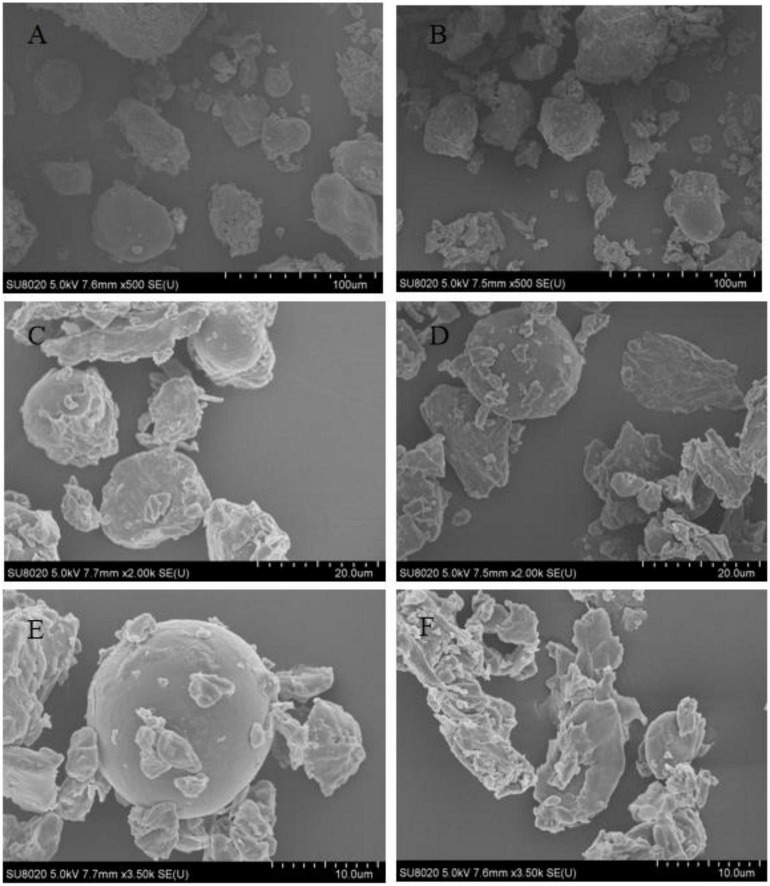
Scanning electron microscope (SEM) images of adzuki bean before and after germination. **(A)** Original adzuki bean (×500), **(B)** Germinated adzuki bean (×500), **(C)** Original adzuki bean (×2000), **(D)** Germinated adzuki bean (×2000), **(E)** Original adzuki bean (×3500), **(F)** Germinated adzuki bean (×3500).

It can be seen from Figure 2 that the whole powder of germinated adzuki bean and original adzuki bean is mainly composed of starch and protein enlarging the whole powder of original adzuki bean and germinated adzuki bean by 500 times, it is found that the whole powder of original adzuki bean has larger particles and less quantity, while the whole powder of germinated adzuki bean not only has more quantity, but also has fewer large particles and more small particles. When the two kinds of starch were enlarged to 2,000 times, the results were the same as those enlarged to 500 times, and the original starch size was about 20 μm, while the germinated starch granules were about 10 μm. The starch granules of the original starch granules were about 20 μm, and the starch granules of the original starch granules of the original starch granules were about 20 μm. In order to make the structure clearer and magnified to 3,500 times, it was found that the starch granules of the original adzuki bean were completely spherical and the surface was smooth and clear, but after germination, the starch granules were broken into many irregular small granules with rough surfaces and protruding grooves. It is possible that endogenous enzymes such as amylase are activated due to germination stress, which makes the polymer of macromolecular substances such as starch degraded and destroys the original polymer structure, so that it can be decomposed into small molecular nutrients to better provide energy for the body (21).

### 3.5. Antioxidant activity of different solvent extracts from germinated adzuki bean

#### 3.5.1. Scavenging ability of extracts from different solvents to DPPH

**FIGURE 3:**
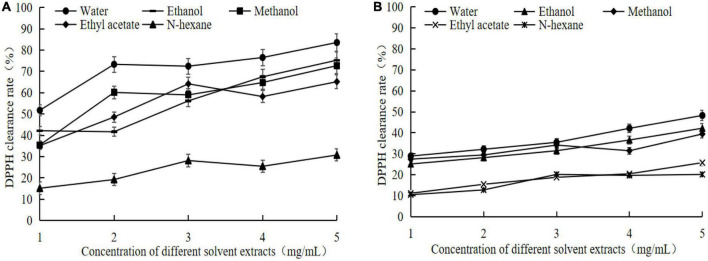
2,20-diphenyl-1-picrylhydrazyl (DPPH) clearance rate of extracts from germinated adzuki bean and adzuki bean. Panel **(A)** is the DPPH scavenging rate of different solvent extracts of germinated adzuki bean, and panel **(B)** is the DPPH scavenging rate of different solvent extracts of adzuki bean.

It can be seen that the DPPH scavenging ability of germinated adzuki bean is obviously improved, compared with that of adzuki bean raw materials, and the change trend of both is consistent, and both increase with the increase of concentration for germinating adzuki bean, the DPPH scavenging ability of different solvent extracts is dose-dependent with the corresponding concentration (Figure 3A). The DPPH scavenging rate of water, ethanol, methanol, ethyl acetate, and hexane extracts were 83.56 ± 5.42%, 75.25 ± 4.16%, 72.69 ± 5.46%, 65.17 ± 4.82%, 30.76 ± 3.36%, when the concentration of germinated adzuki bean extracts was 5 mg/mL, which was 1.731. 781.822. 591.52 times higher than that of raw adzuki bean extracts, respectively (Figure 3B). The DPPH scavenging ability of the water extracts was significantly higher than that of other solvent extracts. The water extracts of adzuki bean contained more GABA, and the scavenging ability of specific free radicals of the raw material adzuki bean was also correspondingly enhanced compared with that of the raw material adzuki bean.

**FIGURE 4:**
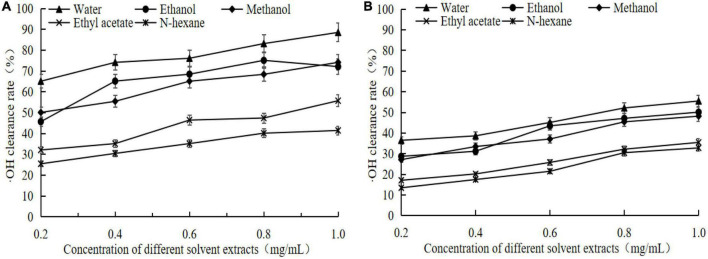
⋅OH clearance of extracts from germinated adzuki bean and adzuki bean. Panel **(A)** is the OH scavenging rate of different solvent extracts of germinated adzuki bean, and panel **(B)** is the OH scavenging rate of different solvent extracts of adzuki bean.

According to Figure 4 showed that the germinated adzuki bean had higher⋅OH radical scavenging rate than raw adzuki bean, in which the scavenging rate of different solvent extracts of germinated adzuki bean is maintained at 88.52–41.48% (Figure 4A), while the scavenging rate of different solvent extracts of raw adzuki bean is maintained at 55.17–32.79% (*P* < 0.05, Figure 4B). When the concentration of extracts was 1 mg/mL, the free scavenging ability of the extracts of germinated adzuki bean and raw adzuki bean to OH was in the order of water extract > ethanol extract > ethanol extract > ethyl acetate extract > N-hexane extract. When the concentration was 1 mg/mL, the scavenging rate of water extract of germinated adzuki bean was 88.52%, which was 1.60 times higher than that of raw adzuki bean. The scavenging effect of ethanol and methanol was not significant, but the scavenging ability of ethyl acetate and N-hexane was the weakest. It can be concluded that germination treatment can significantly increase the antioxidant capacity of adzuki bean, and the best antioxidant effect of water extract may be due to the high content of active components in water extract and good scavenging effect of OH and DPPH.

## 4. Discussion

A wide range of phytochemical changes are induced during germination, which results in a dynamic and complex nutrient flow including nutrient re-mobilization, degradation and accumulation (22). During germination, the activity of protease, amylase, lipase, and other hydrolases is often enhanced. Under the action of these endogenous enzymes, nutrient storage substances such as protein, fat and carbohydrate are degraded into simpler forms, such as small molecular sugars, amino acids, fatty acids, and other substances, thus increasing the accessibility, bioavailability and digestibility of nutrients (23). In this study, the contents of protein, fat and starch in germinated adzuki bean were significantly lower than those of adzuki bean, but the mineral elements of Na, Ca, Mg were significantly higher than those of adzuki bean. Because the stored nutrients are consumed as energy and some of them are decomposed into small molecules by endogenous enzymes, this is consistent with the induction and activation of nutrition mobilization system (24). As for fatty acids, the total amount of fatty acids in adzuki bean decreased significantly after germination, but the types of fatty acids were not affected. Further analysis showed that short chain fatty acids such as caproic acid, caprylic acid, decanoic acid, and lauric acid increased significantly (*P* < 0.05), and the content of linoleic acid and linolenic acid increased by 11.63 and 25.46%, respectively compared with that before germination. It has been reported that oleic acid can be converted into linoleic acid by the action of 12-desaturase and linoleic acid can be converted into DHA by multi-step metabolism. This was confirmed by the increase of EPA, DHA content in germinated adzuki bean in this study.

The decrease of total fatty acids in germinating adzuki bean may be due to the conversion of fatty acids into other new substances under the action of hydrolase, on the other hand, fatty acids provide energy for germination, which is a physiological metabolism and can be used to maintain stress growth. It is worth noting that the total amino acid content of adzuki bean increased 4.43 times compared with that of adzuki bean under germination stress, and the other 21 amino acids increased significantly except homocysteine, among which the three amino acids, was amide, glutamate and arginine, had the highest content and contributed significantly to the total amino acid content. The accumulation of amino acids during the germination of adzuki bean may be due to the activation of protease, which greatly promotes the modification and degradation of stored proteins, and also promotes the metabolism and structural transformation of proteins by encouraging the formation of shorter polypeptides and amino acids (25). At the same time, the attack of enzymes on proteins will also lead to more flexible structural chains of proteins, thus increasing the sensitivity of proteins and being easy to be attacked by proteases (26). Many factors contributed to the increase of amino acids in germinated adzuki bean. The results showed that vacuum combined with MSG had the greatest effect on amino acid content of germinated adzuki bean. Previous studies also confirmed that germination not only increased amino acid content, but also caused significant changes in the composition of free amino acid (27, 28).

The changes of microstructure of adzuki bean before and after germination were better explained by the transformation of protein and amino acid during germination. The microstructure of adzuki bean powder before germination showed that starch granules distributed uniformly and gathered more regularly in protein network structure, while after germination, there were more irregular fragments, and the aggregation of starch granules decreased and the pore structure increased and became coarser. This is similar to the mechanism of amino acid increase in the early stage. The cascade reaction induced by protease attack resulted in the loosening of protein structure and the improvement of protein flexibility, which made the combination of protein and starch no longer tight (20, 29), which led to the difference of microstructure before and after germination of adzuki bean. In a word, the nutritional characteristics of germinated adzuki bean under vacuum combined with MSG stress changed significantly, and the general trend was to be more conducive to increasing the nutritional value of adzuki bean, regulating physiological activities and benefiting human health.

Significant changes in nutritional characteristics of germinated adzuki bean will inevitably lead to changes in physiological and functional characteristics. The antioxidant effect of GABA-rich adzuki bean was evaluated by *in vitro* DPPH radical and⋅OH radical scavenging ability. The results showed that the extracts of germinated adzuki bean showed higher scavenging ability of DPPH and⋅OH radicals than that of raw adzuki bean. It has been reported that germination can increase the antioxidant activity of soybean brown rice and show cytoprotective effect, which is mainly due to its antioxidant polypeptides and glycoproteins (30, 31). Deng et al. (32) eported that GABA can capture the intermediate of lipid peroxidation and react with malondialdehyde, which is a good antioxidant. Other studies have proved that GABA has better antioxidant capacity than positive control butylated hydroxyanisole (BHA) in scavenging DPPH and superoxide radicals (33). In this study, GABA accounted for an important proportion of the total germinated adzuki bean water matter, which also suggested that the accumulation of GABA contributed to the improvement of antioxidant activity of its germinated adzuki bean.

## 5. Conclusion

In summary, the total amount of fatty acids decreased and the total amount of amino acids increased significantly after the adzuki bean germination. The starch granules of the germinated adzuki bean became smaller and coarser. The general trend is that it is more conducive to increasing the nutritional value of adzuki bean and developing in the direction of human health. The antioxidant activity of GABA-rich adzuki bean was significantly better than that of raw adzuki bean. It can be used as the preferred object for the development of natural antioxidant substances.

## Data availability statement

The original contributions presented in this study are included in this article/supplementary material, further inquiries can be directed to the corresponding author.

## Author contributions

XJ was responsible for the conceive and design the experiments. QX and JZ performed the detection of basic nutrition index. ZL was responsible for directing the analysis of amino acid data. HT and DC were responsible for guiding the detection of antioxidant indicators. DZ handled supervision throughout research and manuscript publishing. All authors have read and approved the final manuscript.
